# Costs associated with depression and obesity among cardiovascular patients: medical expenditure panel survey analysis

**DOI:** 10.1186/s12913-021-06428-x

**Published:** 2021-05-06

**Authors:** Felipe Saia Tápias, Victor Henrique Oyamada Otani, Daniel Augusto Corrêa Vasques, Thais Zelia Santos Otani, Ricardo Riyoiti Uchida

**Affiliations:** University of Medical Sciences of Santa Casa de São Paulo, Dr. Cesário Motta Jr. Street, 61, São Paulo, SP 01221-020 Brazil

**Keywords:** Depression, Cost, Cardiovascular conditions, Obesity

## Abstract

**Background:**

There is a lack of information on the cost of depression associated with metabolic syndrome and cardiovascular diseases in the literature.

**Methods:**

We evaluated the synergistic effects of depression and obesity on total expenditures for cardiovascular conditions using data from the Medical Expenditure Panel Survey (MEPS) database. We analyzed MEPS data from 1996 to 2017 comprising adult cardiovascular subjects. We categorized individuals following a combination of International Classification of Diseases ICD-9-CM and ICD-10 codes, and depression symptoms as evaluated using the Patient Health Questionnaire-2 (PHQ-2) depression screening tool. Our sample comprised cardiovascular patients aged 18 years and older, with a body mass index (BMI) between 18.5 and 60. Our study comprised unweighted sample of 96,697 (weighted sample of 938,835,031) adults, a US-nationwide representative sample of cardiovascular disease patients. The four response categories were: no depression; unrecognized depression; asymptomatic depression; and symptomatic depression. Our evaluated outcomes were total annual healthcare expenditures, including dental, emergency room, hospital outpatient, hospital inpatient, office-based, prescription, and home health care expenses.

**Results:**

Asymptomatic and symptomatic depression was more frequent among obese individuals than in individuals with a normal BMI (*p* <  0.001). Total expenditure was highest among symptomatic depression individuals (17,536) and obese (9871) with cardiovascular disease. All the expenditure outcomes were significantly higher among symptomatic depression individuals than those without depression (*p* <  0.001), except for dental costs. All healthcare expenditures associated with obesity were higher compared to individuals with normal BMI with *p* <  0.001, except for emergency and home healthcare costs. Most importantly, among obese individuals, all healthcare expenditures were significantly higher (*p* <  0.001) in those with symptomatic depression than those without depression, except for dental costs, where the difference was not significant (0.899). Therefore, obesity and depression entail increased expenses in patients with cardiovascular disease.

**Conclusions:**

We found incremental expenditures among unrecognized, asymptomatic, and symptomatic depressed individuals with obesity compared to non-depressed, non-obese subjects. However, these are preliminary results that should be further validated using different methodologies.

## Background

Depression is a mental disorder with a significant disease burden affecting people in all communities across the world. The worldwide prevalence of depressive episodes is approximately 3.5% [1] with over 17.3 million individuals (7.1%) diagnosed with major depressive disorder in the US in 2017, this prevalence being higher among individuals self-identified as multi-racial (11.3%) [2]. One out of every five patients with cardiovascular disease is diagnosed with major depressive disorder [3, 4]. Besides, depression is the leading cause of morbidity and low quality of life among cardiovascular patients [5]. Higher expenses have been reported among depressed individuals with cardiovascular conditions, compared to non-depressed subjects. In one study, depressed women had adjusted annual cardiovascular costs $1550 to $3300 higher than not depressed women [6]. Depression was also associated with a 15–53% increase in five-year cardiovascular costs [6].

A review study confirms a common link between depression and obesity as obese individuals are 32% more likely to have depression than the general population [7]. Also, the severity and outcomes of depression associated with obesity are related to several adverse conditions, including hypertension and coronary heart disease, ultimately increasing mortality rates [8]. Depression leads to significant disease and financial burden, with an estimated total cost of $210.5 billion in 2010 [9]. Comorbidities associated with depression contribute 62% to this economic burden [9]. Regardless of the strong association between metabolic syndrome and depression, there is a paucity of information on the cost of this relationship.

Obesity alone accounts for 2 to 8% of the total healthcare expenditure across different countries [10]. In Canada, obesity costs 1.27–11.08 billion Canadian dollars annually [11]. Metabolic syndrome can be even more expensive, costing the European Union around 160 billion euros/year [12]. Previous studies have reported high healthcare costs in obesity driven by the presence of depression and comorbidity [13, 14]. Nevertheless, these studies have not estimated costs using a nationally representative sample, evaluated the difference among depression severity levels, or assessed the role of obesity in cardiovascular conditions.

Given this gap in the literature, our objective was to determine the expenditures of depression among individuals with associated cardiovascular disease and obesity.

## Methods

### Study design

We extracted information from the Medical Expenditure Panel Survey (MEPS) database. We describe this study following RECORD (REporting of studies Conducted using Observational Routinely-collected Data) [15], an extension of the STROBE (STrengthening the Reporting of OBservational studies in Epidemiology) guidelines [16].

### Ethics

The Institutional Review Board of the Santa Casa de São Paulo School of Medicine, Brazil, approved our study.

### Setting

We obtained data from MEPS from 1996 to 2017 [17]. This database represents estimates of health care utilization and expenses for the non-institutionalized United States civilian population, including household, medical provider, and insurance costs. Estimates of respondents’ health status, demographic and socio-economic characteristics, access to care, and satisfaction with health care can be generated for individuals, families, and select population subgroups. Each MEPS data collection wave consists of five rounds of interviews covering two full calendar years, creating a pooled cross-sectional sample.

### Participants

Inclusion criteria comprised patients aged 18 years and older with a body mass index (BMI) greater than 18.5 and less than 60, and diagnosed with cardiovascular conditions identified by the International Classification of Disease Version-9-Clinical Modification (ICD-9-CM) from 1996 to 2015 and International Classification of Diseases, Tenth Revision (ICD-10) diagnosis codes from 2016 to 2017 (Table 6 in Appendix). We used BMI data from 2001 until 2015, as the MEPS did not include a BMI variable for other study research periods. We excluded all pregnant women with either heart disease or obesity.

### Outcomes

Outcomes of interest were total healthcare expenditures for the calendar year, including dental, emergency room, hospital outpatient, hospital inpatient, office-based, prescription, and home healthcare expenses. MEPS defines expenditure as the total amount of payments for healthcare services provided during the year. These fees comprise Medicare, Medicaid, out-of-pocket payments, payments by insurance, and any other sources.

### Predictors

Our predicting variables were the four mutually exclusive depression categories created from a combination of the clinical signs of depression identified by ICD-9-CM and ICD-10 codes, and depression symptoms evaluated using the PHQ-2 questionnaire [18]. We identified clinical signs of depression through the ICD-9-CM codes 296 (episodic affective disorders), 309 (adjustment reaction), and 311 (depressive disorder, not elsewhere classified) and the corresponding ICD-10 diagnostic codes (F34, F34.9, F43.2, and F32.9). The PHQ-2 is a two-question self-report questionnaire that makes up part of the MEPS survey using a cutoff of greater than or equal to three and presenting a sensitivity of 83 and 92% specificity for screening major depression [19]. Next, we created two dummy variables using the ICD-9/ICD-10 diagnosis and a PHQ-2 cut-point of ≥3 for each patient with cardiovascular conditions or obesity on the MEPS.

The resulting depression categories were:

1. No depression, defined as individuals with neither depressive symptoms as determined by the PHQ-2 nor ICD-9/ICD-10 diagnosis of depression;
2. Unrecognized depression, defined as individuals with PHQ-2 scoring positive for depressive symptoms but without ICD-9/ICD-10 diagnosis of depression;
3. Asymptomatic depression, defined as individuals with an ICD-9/ICD-10 diagnosis of depression but may not have been deemed positive with PHQ-2 depressive symptoms or may be undergoing treatment and have no more symptoms; and
4. Symptomatic depression, which we determined as individuals with both PHQ-2 depressive symptoms and clinical depression by ICD-9/ICD-10 diagnosis.

These categories do not represent the severity of depression, but the presence or absence of diagnosis or the presence or absence of symptoms.

### Potential confounders

We selected potential confounders using evidence from the literature and clinical judgment because this combination performs better than isolated clinical and evidence-based criteria [20]. We selected age, gender, employment (employed and not employed), health insurance (private, public, and uninsured), income level (USD), marital status (married, widowed, divorced, separated, and never married), smoking status (smoker and non-smoker), and race (White, Black, Asian, Hispanic, and other) [18, 21].

### Data access and cleaning methods

We computed the expenditures of cardiovascular patients for the following categories: office (sum of all variables related to office visits for each year), outpatient (sum of all variables related to outpatient visits for each year), emergency (sum of all variables related to emergency room visits for each year), inpatient (sum of all variables related to the inpatient hospital stay and zero-night inpatient stay for each year), prescription (sum of all variables related to total RX for each year), dental (sum of all variables related to dental care for each year), home healthcare (sum of all variables related to home health agency and home health non-agency for each year), and others (sum of all variables related to glasses and lenses and other equipment or supplies for each year). Total expenditures were calculated as the sum of all previously mentioned categories. As MEPS presents multiple datasets split by year, we combined these datasets by the DUPERSID, which is the sample person identifier.

Next, we applied our inclusion criteria (> 18 years, and BMI > 18.5 and < 60). We categorized the remaining sample by BMI (< 25 as normal, ≥25 and < 30 as overweight, and ≥ 30 as obese), depression diagnosis, and PHQ2 scores. Finally, we adjusted all expenditure variables for inflation to a common 2019 US dollar value following the consumer price index (CPI) [22].

### Statistical methods

We visually inspected the data for the frequency, percentage, and near-zero variance for categorical variables (BMI, marital status, health insurance, and employment). We also evaluated the distribution for numeric variables (age, healthcare expenditures including dental, emergency room, hospital outpatient, hospital inpatient, office-based, prescription, and home health care expenses), and missing value patterns [23]. Near-zero variance occurs when a categorical variable has a low frequency of unique values over the sample size, i.e., the variable is almost constant, and we addressed it by combining different variable categorizations. We made comparisons through a standardized difference, i.e., the difference in means or proportions divided by the pooled standard deviation.

We used a series of survey-weighted generalized linear regression models to evaluate the impact of depression and obesity on the financial expenditures among individuals with cardiovascular conditions. In our models, the outcome variable was the financial expenditures and the predictor variables constituted the four depression categories, and three BMI categories in subjects with cardiovascular disease. Since the expenditure variables did not present a normal distribution, all models were run with log-transformed variables and then subsequently exponentiated so that results could be clinically interpretable. Thus, all results were reported as predicted medians (instead of predicted means) with 95% Confidence Intervals [24]. Our models were evaluated using Root Squared (R-squared) metric. The R squared is the coefficient of determination and indicates the percentage of variance of outcome variable explained by the predictor variables of the model. We used the R-squared as a goodness-of-fit indicator, higher values close to 1 represents a good item fit, because it indicates that the predictors are able to explain most of the total variance of the outcome variable. The R-squared value ranges from 0 to 1 with 1 being a perfect predictive accuracy. We also started from a full model while progressively deleting variables while using log-likelihood tests to reach the most parsimonious model. We interpreted results as statistically significant when the confidence intervals did not overlap among different estimates and with *p*-values < 0.001 [24]. We performed multiple testing correction using the Benjamini-Hochberg’s method [25] to control for false discovery rate (FDR).

We adjusted our analyses for weights (multipliers relating the sample to the total population), primary sampling units (sample aggregates), and strata (subpopulations) [26, 27]. Such adjustments enable the inferences to be extended to a larger population [26, 27]. We report frequencies as the number of individuals in the target population and adjust our confidence intervals to the population rather than to our study sample. Therefore, our results represent the synergistic effects of depression and obesity on total expenditures for cardiovascular conditions in the US population.

While modeling the data, we conducted stratified analyses involving individuals with a BMI ≥30. We performed all analyses using the R language (R version 4.0.2) [28].

## Results

### Participants

Our original unweighted study sample consisted of 96,697 adults with cardiovascular conditions. After adjusting for sampling weights, clustering, and stratification design, our inferred study population included 938,835,031 subjects. Table 1 describes the weighted study population stratified by a depression diagnosis. The sample presented a mean age of 61.5 years, of which 77.2% had no depression, 5.38% had unrecognized depression, 13% had asymptomatic depression, and 4.35% had symptomatic depression. Individuals with symptomatic depression were younger than the others. A lower proportion of individuals with symptomatic depression were married (40% vs. 61, 48, and 51% in other categories) whereas depression was higher among divorced (26% vs. 12, 16, and 18%), separated (5% vs. 1, 3, and 3%), and never married (14% vs. 8, 12, and 10%). Dependence on public insurance was more likely in individuals with symptomatic depression (48%) than in those with asymptomatic depression (30%), unrecognized depression (46%), and no depression (26%). Non-depressed people were more likely to have any private insurance than symptomatic individuals (68% vs. 42%). There was a decreasing trend in wage levels by depressive state, people without depression presenting the highest salary (23,386), and symptomatic patients the lowest (8919). Individuals with symptomatic depression were more likely to be obese (54%), non-employed (75%), and smoker (32%). Total expenditure was highest for individuals with symptomatic depression (17,536 USD) and lowest for those without depression (8402 USD).

**Table 1:** Sample characteristics categorized by the diagnosis of depression among individuals with cardiovascular conditions presented as weighted sample size (Unweighted *n* = 96,697)

Variable	Total *n* = 938,835,031	No depression *n* = 724,868,599	Unrecognized depression *n* = 50,539,118	Asymptomatic depression *n* = 122,568,131	Symptomatic depression *n* = 40,859,184	***p***
Age	61.5 ± 0.1	62.0 ± 0.1	61.6 ± 0.3	60.2 ± 0.2	57.0 ± 0.3	< 0.001
Female	118,073,895 (12.6%)	88,921,992 (12.3%)	6,149,388 (12.2%)	17,136,457 (14%)	5,866,058 (14.4%)	< 0.001
Marital status						< 0.001
*Married*	548,568,787 (58.4%)	443,957,776 (61.2%)	24,654,404 (48.8%)	63,380,151 (51.7%)	16,576,456 (40.6%)	
*Widowed*	145,934,265 (15.5%)	111,472,105 (15.4%)	8,890,367 (17.6%)	20,172,914 (16.5%)	5,398,878 (13.2%)	
*Divorced*	133,376,145 (14.2%)	91,872,866 (12.7%)	8,561,595 (16.9%)	22,246,025 (18.1%)	10,695,659 (26.2%)	
*Separated*	20,487,099 (2.18%)	12,641,485 (1.74%)	1,952,166 (3.86%)	3,769,402 (3.08%)	2,124,046 (5.2%)	
*Never married*	90,453,865 (9.63%)	64,909,495 (8.95%)	6,480,585 (12.8%)	12,999,639 (10.6%)	6,064,145 (14.8%)	
Health insurance						< 0.001
*Any private*	616,141,312 (65.6%)	497,948,903 (68.7%)	22,175,659 (43.9%)	78,549,568 (64.1%)	17,467,182 (42.7%)	
*Public only*	268,783,570 (28.6%)	188,172,701 (26%)	23,673,089 (46.8%)	37,290,848 (30.4%)	19,646,932 (48.1%)	
*Uninsured*	53,910,150 (5.74%)	38,746,994 (5.35%)	4,690,370 (9.28%)	6,727,715 (5.49%)	3,745,071 (9.17%)	
Employment						< 0.001
*Employed*	429,783,368 (45.8%)	354,917,968 (49.1%)	13,885,184 (27.6%)	51,029,996 (41.8%)	9,950,221 (24.4%)	
*Not employed*	505,727,921 (53.9%)	367,278,193 (50.9%)	36,472,070 (72.4%)	71,148,218 (58.2%)	30,829,440 (75.6%)	
Wage	21,338 ± 266	23,386 ± 290	9876 ± 428	18,090 ± 482	8919 ± 478	< 0.001
Smoke	137,182,754 (14.6%)	88,298,836 (12.2%)	12,290,545 (24.3%)	23,132,088 (18.9%)	13,461,284 (32.9%)	< 0.001
Body Mass Index						< 0.001
*Normal*	213,354,729 (22.7%)	169,372,686 (23.4%)	11,865,490 (23.5%)	24,541,148 (20%)	7,575,406 (18.5%)	
*Overweight*	333,797,512 (35.6%)	267,335,467 (36.9%)	16,193,934 (32%)	39,114,031 (31.9%)	11,154,080 (27.3%)	
*Obese*	391,682,790 (41.7%)	288,160,446 (39.8%)	22,479,693 (44.5%)	58,912,952 (48.1%)	22,129,699 (54.2%)	
Office expenditure	2528 ± 32	2331 ± 35	2913 ± 113	3203 ± 72	3524 ± 130	< 0.001
Outpatient expenditure	859 ± 28	792 ± 29	931 ± 80	1037 ± 71	1306 ± 141	< 0.001
Emergency room expenditure	334 ± 9	288 ± 10	518 ± 34	404 ± 20	613 ± 49.5	< 0.001
Inpatient expenditure	2210 ± 55	1852 ± 54	4173 ± 323	2772 ± 142	4444 ± 288	< 0.001
Prescription expenditure	3089 ± 40	2579 ± 33	3925 ± 116	4711 ± 157	6243 ± 310	< 0.001
Dental expenditure	364 ± 6	369 ± 6	221 ± 16	411 ± 16	313 ± 19	< 0.001
Home healthcare expenditure	500 ± 29	367 ± 22	1273 ± 147	756 ± 128	1139 ± 114	< 0.001
Other expenditure	185 ± 5	164 ± 4	234 ± 32	247 ± 14	311 ± 32	< 0.001
Total expenditure	9712 ± 106	8402 ± 93	13,888 ± 445	13,130 ± 294	17,536 ± 658	< 0.001

We present all numeric or continuous variables as mean ± standard deviation and display categorical variables as frequency (percentages). All variable results are presented as weighted sample size. The measurement units for the wage and expenditure variables is USD

We found that 35.6% of individuals with cardiovascular conditions were overweight, and 41.7% were obese. Obese individuals were more likely to be young (mean age of 58 years old), female (13.3%), employed (51.3%), and with a high salary (mean salary of 22,797 USD). Obese individuals presented a higher percentage of unrecognized depression (5.7%), asymptomatic depression (15%), and symptomatic depression (5.6%). Obese subjects reported higher outpatient (984 USD), prescription (3235 USD), and total (9871 USD) mean expenditures (Table 2).

**Table 2:** Sample characteristics stratified by BMI presented as weighted sample size (Unweighted n = 96,697)

Variable	Total *n* = 938,835,031	Normal *n* = 213,354,729	Overweight *n* = 333,797,512	Obese *n* = 391,682,790	***p***
Age	61.5 ± 0.1	65.9 ± 0.2	62.9 ± 0.2	58.0 ± 0.2	< 0.001
Female	118,073,895 (12.6%)	27,062,571 (12.7%)	38,864,259 (11.6%)	52,147,065 (13.3%)	< 0.001
Marital status					< 0.001
*Married*	548,568,787 (58.4%)	113,574,344 (53.2%)	204,921,087 (61.4%)	230,073,356 (58.7%)	
*Widowed*	145,934,265 (15.5%)	48,649,769 (22.8%)	50,657,870 (15.2%)	46,626,626 (11.9%)	
*Divorced*	133,376,145 (14.2%)	28,249,208 (13.2%)	45,107,797 (13.5%)	60,019,140 (15.3%)	
*Separated*	20,487,099 (2.18%)	3,851,026 (1.8%)	6,186,703 (1.85%)	10,449,370 (2.67%)	
*Never married*	90,453,865 (9.63%)	19,030,383 (8.92%)	26,914,200 (8.06%)	44,509,282 (11.4%)	
Health insurance					< 0.001
*Any private*	616,141,312 (65.6%)	132,435,177 (62.1%)	224,022,303 (67.1%)	259,683,832 (66.3%)	
*Public only*	268,783,570 (28.6%)	71,304,323 (33.4%)	92,063,226 (27.6%)	105,416,020 (26.9%)	
*Uninsured*	53,910,150 (5.74%)	9,615,230 (4.51%)	17,711,982 (5.31%)	26,582,938 (6.79%)	
Employment					< 0.001
*Employed*	429,783,368 (45.8%)	75,662,429 (35.6%)	153,705,062 (46.2%)	200,415,877 (51.3%)	
*Not employed*	505,727,921 (53.9%)	136,759,689 (64.4%)	179,001,926 (53.8%)	189,966,305 (48.7%)	
Wage	21,338 ± 266	16,621 ± 396	22,640 ± 386	22,797 ± 350	< 0.001
Smoke	137,182,754 (14.6%)	37,266,102 (17.5%)	46,438,935 (13.9%)	53,477,717 (13.7%)	< 0.001
**Depression**					< 0.001
***No depression***	724,868,599 (77.2)	169,372,686 (79.4%)	267,335,467 (80.1%)	288,160,446 (73.6%)	
***Unrecognized depression***	50,539,118 (5.38%)	11,865,490 (5.56%)	16,193,934 (4.85%)	22,479,693 (5.74%)	
***Asymptomatic depression***	122,568,131 (13.1%)	24,541,148 (11.5%)	39,114,031 (11.7%)	58,912,952 (15%)	
***Symptomatic depression***	40,859,184 (4.35%)	7,575,406 (3.55%)	11,154,080 (3.34%)	22,129,699 (5.65%)	
Office expenditure	2323 ± 30	2433 ± 59	2228 ± 47	2344 ± 39	0.019
Outpatient expenditure	901 ± 29	853 ± 54	829 ± 36	984 ± 44	0.078
Emergency room expenditure	350 ± 9	387 ± 25	318 ± 17	357 ± 13	0.013
Inpatient expenditure	2317 ± 58	2410 ± 124	2239 ± 95	2333 ± 76	0.266
Prescription expenditure	2835 ± 39	2584 ± 56	2526 ± 36	3235 ± 72	< 0.001
Dental expenditure	382 ± 6	400 ± 11	398 ± 9	358 ± 8	< 0.001
Home healthcare expenditure	520 ± 30	862 ± 89	378 ± 23	456 ± 38	< 0.001
Other expenditure	194 ± 5	219 ± 11	197 ± 8	179 ± 5	< 0.001
**Total expenditure**	**9447 ± 109**	**9743 ± 232**	**8760 ± 143**	**9871 ± 155**	**< 0.001**

We present all numeric or continuous variables as mean ± standard deviation and display categorical variables as frequency (percentages). All variable results are presented as weighted sample size. The measurement units for the wage and expenditure variables is USD

**Fig. 1:**
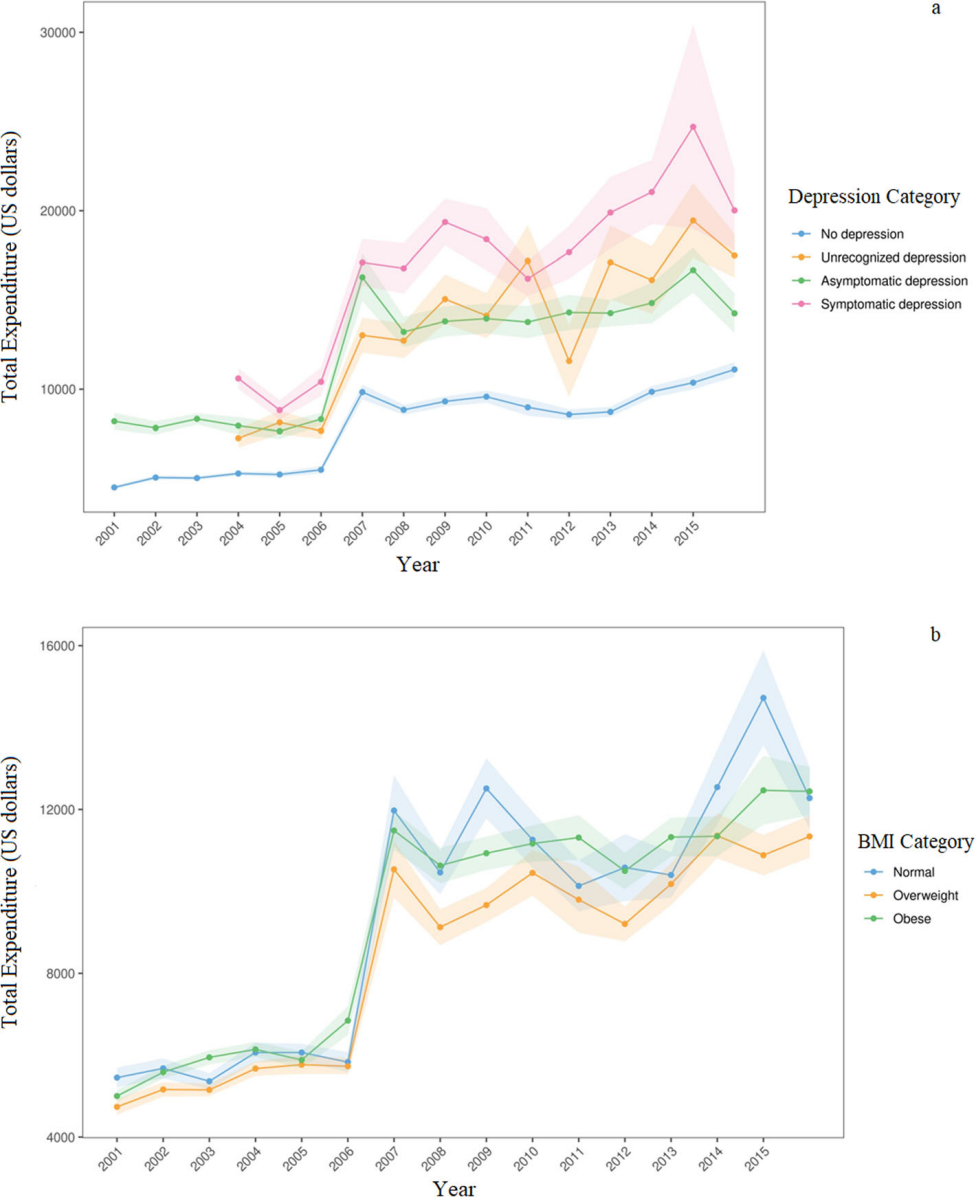
**a** Total expenditure (in USD) among individuals with cardiovascular conditions from 2001 to 2015 stratified by depression category. **b** Total expenditure (in USD) among individuals with cardiovascular conditions from 2001 to 2015 stratified by BMI category

Figure 1a evaluates the association between total expenditure and depression category among cardiovascular individuals. Despite a decrease over 2004–2006 and 2009–2011, symptomatic depressed individuals demonstrated higher sustained expenditures, with a gradual increase over 2011–2015. Unrecognized depression subjects presented low expenditures over the 2011–2012 year, but incremental increases towards 2015. The total expenditure in non-depressed and asymptomatic subjects remained stable. Besides, no-depression individuals reported lower expenditures than those who were unrecognized or asymptomatic.

When evaluating the association between total expenditure and BMI categories, we found that total annual expenditure among cardiovascular individuals did not vary substantially between BMI categories across 2001–2005. Since 2006, individuals with normal BMI demonstrated expenditures similar to but higher than obese towards the year 2015 (Fig. 1b).

Table 3 displays the association between healthcare expenditure and depression, displayed as predicted medians with 95% confidence intervals (CI) in parenthesis. There was an incremental range of costs for office, outpatient, prescription drugs, other, and total expenditures that were significantly different among unrecognized, asymptomatic, and symptomatic depression compared to not-depressed subjects. All expenditure outcomes were statistically highest among symptomatic depression individuals than those without depression, except for dental costs. Office, emergency, inpatient, prescription, dental, home healthcare, other, and total expenditures were significantly higher among individuals with unrecognized and asymptomatic depression than those with no depression (*p* <  0.001). Table 7 in Appendix presents the R-square measures for the amount of variance of each model.

**Table 3:** Predicted medians (in USD) for normal individuals, individuals with unrecognized, asymptomatic and symptomatic depression

Expenditure	No depression [median (95% CI^a^)]	Unrecognized depression [median (95% CI^a^)]	***p***	Asymptomatic depression [median (95% CI^a^)]	***p***	Symptomatic depression [median (95% CI^a^)]	***p***
Office	**306 (293, 319)**	**394 (362, 428)**	**< 0.001**	**562 (530, 596)**	**< 0.001**	**755 (690, 826)**	< 0.001
Outpatient	**4.58 (4.29, 4.89)**	**5.51 (4.82, 6.29)**	**0.007**	**8.07 (7.05, 9.24)**	**< 0.001**	**10.8 (9.07, 12.9)**	< 0.001
Emergency room	**3.03 (2.89, 3.16)**	**6.23 (5.52, 7.04)**	**< 0.001**	**4.47 (4.06, 4.94)**	**< 0.001**	**9.05 (7.82, 10.5)**	< 0.001
Inpatient	**2.13 (2.05, 2.21)**	**4.51 (3.94, 5.15)**	**< 0.001**	**3.01 (2.77, 3.27)**	**< 0.001**	**6.02 (5.22, 6.94)**	< 0.001
Prescription	**545 (525, 566)**	**795 (742, 851)**	**< 0.001**	**1213 (1148, 1282)**	**< 0.001**	**1794 (1655, 1944)**	< 0.001
Dental	**7.83 (7.44, 8.24)**	**3.94 (3.6, 4.32)**	**< 0.001**	**9.13 (8.38, 9.95)**	**< 0.001**	**6.43 (5.65, 7.33)**	< 0.001
Home healthcare	**1.44 (1.4, 1.48)**	**2.48 (2.21, 2.77)**	**< 0.001**	**1.97 (1.84, 2.11)**	**< 0.001**	**2.73 (2.43, 3.07)**	0.005
Other	**3.53 (3.41, 3.65)**	**4.39 (4, 4.81)**	**< 0.001**	**5.84 (5.44, 6.29)**	**< 0.001**	**7.57 (6.62, 8.66)**	< 0.001
Total	2075 (2005, 2147)	3232 (3035, 3442)	< 0.001	3875 (3697, 4061)	< 0.001	5844 (5458, 6258)	< 0.001

^a^*CI* Confidence Interval

When evaluating the relationship between healthcare expenditure and BMI categories, we found that all the expenditure outcomes, including total expenditure, were significantly higher among obese individuals than those with normal BMI, except for emergency room and home healthcare expenditure (*p* <  0.001) (Table 4). Compared to individuals with normal BMI, prescription and home healthcare expenditures were significantly higher among overweight individuals (*p* <  0.001). Table 8 in Appendix presents the R-square measures for the amount of variance of each model.

**Table 4:** Association between healthcare expenditure (in USD) and body mass index

Expenditure	Normal [median (95% CI^a^)]	Overweight [median (95% CI^a^)]	***p***	Obese [median (95% CI^a^)]	***p***
Office	317 (300, 336)	313 (299, 328)	0.652	407 (388, 428)	< 0.001
Outpatient	4.49 (4.11, 4.9)	4.63 (4.3, 5)	0.559	6.25 (5.75, 6.79)	< 0.001
Emergency room	3.71 (3.47, 3.98)	3.28 (3.09, 3.48)	0.002	3.76 (3.58, 3.96)	0.713
Inpatient	2.36 (2.21, 2.51)	2.28 (2.17, 2.41)	0.549	2.68 (2.58, 2.8)	< 0.001
Prescription	486 (461, 512)	578 (553, 604)	< 0.001	854 (819, 890)	< 0.001
Dental	8.68 (8.05, 9.35)	7.9 (7.46, 8.37)	0.029	6.76 (6.39, 7.15)	< 0.001
Home healthcare	1.74 (1.66, 1.83)	1.44 (1.39, 1.48)	< 0.001	1.68 (1.63, 1.74)	0.319
Other	3.71 (3.5, 3.94)	3.65 (3.49, 3.81)	0.652	4.39 (4.2, 4.59)	< 0.001
Total	2129 (2029, 2233)	2188 (2107, 2272)	0.227	2858 (2750, 2969)	< 0.001

^a^*CI* Confidence Interval

### Subgroup analysis

Table 5 shows that total expenditure was significantly higher among obese individuals with symptomatic depression (6246; 95% CI, 5716-6825) than those without depression (2271; 95% CI, 2178-2369). Except for dental expenditure, all other costs increased (*p* <  0.001). Compared to individuals with no depression, office, outpatient, emergency, inpatient, prescription, home healthcare, other, and total expenditures were significantly higher among individuals with asymptomatic depression (*p* <  0.001). Table 9 in Appendix presents the R-square measures for the amount of variance of each model.

**Table 5:** Predicted median expenditures (in USD) among obese patients with cardiovascular conditions stratified by depression levels

Expenditure	No depression [median (95% CI^a^)]	Unrecognized depression [median (95% CI^a^)]	***p***	Asymptomatic depression [median (95% CI^a^)]	***p***	Symptomatic depression [median (95% CI^a^)]	***p***
Office	319 (302, 337)	414 (369, 464)	< 0.001	617 (573, 664)	< 0.001	846 (759, 944)	< 0.001
Outpatient	5.02 (4.59, 5.5)	5.39 (4.44, 6.53)	0.505	8.64 (7.32, 10.2)	< 0.001	11.9 (9.32, 15.2)	< 0.001
Emergency room	3.3 (3.14, 3.47)	5.95 (5.06, 7)	< 0.001	4.51 (3.93, 5.17)	< 0.001	8.89 (7.3, 10.8)	< 0.001
Inpatient	2.22 (2.12, 2.32)	3.95 (3.28, 4.75)	< 0.001	2.92 (2.62, 3.26)	< 0.001	5.46 (4.38, 6.8)	< 0.001
Prescription	632 (602, 662)	913 (832, 1001)	< 0.001	1424 (1330, 1524)	< 0.001	2042 (1828, 2282)	< 0.001
Dental	6.62 (6.2, 7.08)	3.7 (3.21, 4.26)	< 0.001	8.11 (7.24, 9.1)	0.002	6.7 (5.68, 7.89)	0.899
Home healthcare	1.45 (1.4, 1.5)	2 (1.74, 2.29)	< 0.001	1.84 (1.69, 2.01)	< 0.001	2.47 (2.16, 2.83)	< 0.001
Other	3.7 (3.53, 3.87)	3.86 (3.42, 4.36)	0.505	5.75 (5.21, 6.34)	< 0.001	8.09 (6.83, 9.58)	< 0.001
Total	2271 (2178, 2369)	3284 (3007, 3586)	< 0.001	4141 (3889, 4410)	< 0.001	6246 (5716, 6825)	< 0.001

^a^*CI* Confidence Interval

## Discussion

Asymptomatic and symptomatic depression were more frequent among obese individuals, corroborating previous evidence on obesity and depression as comorbid conditions [29]. Longitudinal studies suggest that obesity can increase the risk of depressive symptoms [30] and that mental health conditions like depression are part of the pathophysiology leading to obesity [31]. Furthermore, social factors add complexity to the depression and obesity causal pathway in cardiovascular patients. For instance, a more significant proportion of obese individuals were uninsured compared to overweight and lean subjects. We could explain this finding based on the differential premiums charged to obese patients by insurers before 2014, as stated in the Affordable Care Act [32, 33].

Similarly, individuals with symptomatic and unrecognized depression presented higher unemployment rates and lacked insurance compared to asymptomatic and non-depressed subjects. In fact, preceding evidence suggests that depression treatment lowers unemployment rates in this population [34]. Moreover, unemployment and poor mental health are associated [35–37].

Previous studies that did not focus on cardiovascular disease reported higher expenses in obese and overweight subjects [38]. These studies hypothesized that higher expenses result from poorer health-related behaviors, outcomes, and healthcare utilization [39, 40]. The presence of comorbidities (such as cardiovascular disease) and depression drive increased healthcare costs in obesity [13].

On the other hand, there is a paradoxical association between being obese and better outcomes in cardiovascular patients [41]. Although bias and confounding factors explain this association to a certain degree [41–43], well-designed research supports the obesity paradox [44]. If obese subjects were genuinely at a lower risk of mortality and complications from cardiovascular diseases, one should expect to find lower expenditures among them. Given that lean individuals may present further conditions that drive increased expenditures, some healthcare services may increase. Likewise, we report higher home healthcare expenditures among non-obese participants. Indeed, studies supporting the obesity paradox are frequent among patients with conditions that increase home healthcare expenditures, including heart failure [45]. However, our results demonstrate higher total expenditure among the obese, prescription costs being its primary driver. These findings support preceding results in non-nationally representative studies [39]. These results also align with the current practice, where physicians offer more aggressive treatment modalities to obese patients [46, 47]. A recent review focused on the role of regional fat in the pathogenesis of cardiovascular disease in obese individuals. Furthermore, the rapidly expanding subgroup of patients with severe obesity (BMI > 40 kg/m^2^) represents additional cardiovascular risks requiring aggressive treatments such as metabolic surgery [48].

We found an incremental spectrum of expenditures among unrecognized, asymptomatic, and symptomatic depressed individuals. Prior results suggest that depression increases the expenditures associated with cardiovascular diseases. For instance, depressed women with coronary artery disease present higher expenses than their non-depressed counterparts [6]. Similarly, it is more expensive to treat patients with simultaneous heart failure and depression [49]. Moreover, patients with physical conditions such as cardiovascular, metabolic, and respiratory diseases or cancer who also had treatment-resistant depression have higher health care resource utilization and costs than patients with these same conditions but not treatment-resistant depression [50]. Indeed, depression was the primary driver of cost among obese patients, only surpassed by cardiovascular-related comorbidities [13]. Our results support the concept that depressive symptoms account for increased total expenditure levels [14, 51]. Recent studies state that comorbid depression has a substantial impact on the healthcare costs and utilization of medical services in patients with diabetes [52], migraines [53], hypertension, cardiac disease, and chronic pain [54]. As a result, depression contributes significantly to health, economic, and societal burdens, with an average per-person medical cost of 3.5 times higher than non-depressed ones [55]. Increased expenses for individuals with unrecognized depression may not be attributed to the increase in spending on medication since this category represents individuals with depressive symptoms and without an ICD-9/ICD-10 diagnosis of depression, and thus without a defined diagnosis, drug treatment would not have started. Therefore, we can see that even the patient without undergoing drug treatment already has an increase in healthcare costs and is not due to polypharmacy. Individuals with symptomatic depression were associated with higher health expenses, and so we can contemplate that with the use of medications, psychotherapy, or other therapies, costs are higher than those with unrecognized depression or asymptomatic depression.

Symptomatic subjects presented the top total, office, and prescription expenditures raising questions regarding their treatment effectiveness. Possible explanations include symptomatic patients not being effectively treated or having low compliance levels with therapy. Depressed subjects exhibit low adherence to interventions relevant for both cardiovascular and obesity management, including rehabilitation [56, 57]. In fact, hypertensive patients with depression were reported to have higher risk of non-adherence [58]. Also, low levels of depression promoted the maintenance of weight loss in obese subjects [59].

Similarly, obese subjects identified depression as a hindrance to weight loss [60]. Even if prescriptions were the primary driver of expenditures in depressed patients, the observed differences were not circumscribed to a single branch of medical expenditures, but spread across the whole healthcare system, once again agreeing with previous reports [51]. These effects remained over time, with a substantial rise for those with symptomatic depression.

Depression-associated expenditures substantially increased between 2011 and 2015. Previous research demonstrated a rise in total expenditures in patients with diabetes and unrecognized depression [18]. However, we observed increased expenditures in symptomatic patients but not in subjects with unrecognized depression. Unrecognized depression prevailed in diabetic patients compared to our population, potentially explaining the expenditure differences. Although increased expenses may result from the rising prevalence of depression [61, 62], changes in the diagnostic criteria may have accounted for potential inflation in the US population of depressed patients [63].

Moreover, changes in structured diagnostic interviews may be responsible for the depression prevalence, as reported in Canada [64], casting doubt on this explanation. Another possibility is that medication, therapy, and other medical services have become more expensive. The finding that antidepressant drug expenditure has increased in the last decade corroborates this idea [65].

## Conclusions

To our knowledge, we report the first assessment of the economic interplay between obesity and depression among individuals with cardiovascular conditions, using a US nationally representative sample. Depression prevailed among the obese. Individuals with asymptomatic depression, unrecognized depression, and symptomatic depression presented greater total costs than those without depression. Total expenditures increased from 2011 to 2015 for all categories, and the expenditures for individuals with symptomatic depression had a growth rate that far exceeds the increase for other categories. Obese patients incur higher total expenditures than subjects with normal BMI, the main expenditure component being prescriptions. This finding suggests that efforts to improve screening and management of depression and obesity among cardiovascular patients help to reduce the health and economic burden. Our study further emphasizes the importance of health policies and prevention programs. These results can support policymakers in the decision-making focusing on cardiovascular disease management, especially cost-effective interdisciplinary treatment approaches for depression associated with metabolic risk factors. Therefore, using existing infrastructure to deliver mental health care might reduce societal and personal healthcare expenses.

Despite filling a relevant literature gap, our study has the limitations usually associated with an observational design. For instance, the association found among depression, obesity, and expenditure does not allow for causal inferences. Causal relationships can be delineated in the future using Bayesian network modeling or propensity scores with a sufficient set of confounders. Moreover, future studies will need to use additional metrics other than BMI (i.e. waist circumference) to best represent patient fitness. Finally, depression may interact with different cardiovascular conditions in ways that we did not explore. Given these limitations, further research to acknowledge and address them with robust analyses is warranted.

## Data Availability

The datasets analyzed during the current study are available in the Medical Expenditure Panel Survey (MEPS) database, a US open access repository. The database can be accessed at www.meps.ahrq.gov/mepsweb/.

## Appendix

**Table 6:** List of included ICD-9 and ICD-10 diagnosis codes

ICD-9	ICD-10
401 Essential hypertension	I10 Essential (primary) hypertension
402 Hypertensive heart disease	I11 Hypertensive heart disease
403 Hypertensive chronic kidney disease	I12 Hypertensive chronic kidney disease
404 Hypertensive heart and chronic kidney disease	I13 Hypertensive heart and chronic kidney disease
405 Secondary hypertension	I15 Secondary hypertension
410 Acute myocardial infarction	I21 Acute myocardial infarction; I22 Subsequent ST elevation (STEMI) and non-ST elevation (NSTEMI) myocardial infarction; I23 Certain current complications following ST elevation (STEMI) and non-ST elevation (NSTEMI) myocardial infarction (within the 28 day period); I24 Other acute ischemic heart diseases
413 Angina pectoris	I20 Angina pectoris
414 Other forms of chronic ischemic heart disease	I25 Chronic ischemic heart disease
427 Cardiac dysrhythmias	I47 Paroxysmal tachycardia; I48 Atrial fibrillation and flutter; I49 Other cardiac arrhythmias
428 Heart failure	I50 Heart failure
429 Ill-defined descriptions and complications of heart disease	I51 Complications and ill-defined descriptions of heart disease
433 Occlusion and stenosis of precerebral arteries	I65 Occlusion and stenosis of precerebral arteries, not resulting in cerebral infarction
434 Occlusion of cerebral arteries	I66 Occlusion and stenosis of cerebral arteries, not resulting in cerebral infarction
435 Transient cerebral ischemia	G45 Transient cerebral ischemic attacks and related syndromes
436 Acute, but ill-defined, cerebrovascular disease	I67 Other cerebrovascular diseases
437 Other and ill-defined cerebrovascular disease	I67 Other cerebrovascular diseases
440 Atherosclerosis	I70 Atherosclerosis
443 Arterial embolism and thrombosis	I74 Arterial embolism and thrombosis
447 Other disorders of arteries and arterioles	I77 Other disorders of arteries and arterioles

**Table 7:** R-square measures for each model for the evaluation of normal individuals, individuals with unrecognized, asymptomatic and symptomatic depression

Expenditure	Unrecognized depression	Asymptomatic depression	Symptomatic depression	R-squared
Office	**0.253 (0.177, 0.328) [*****p*** **< 0.001]**	**0.609 (0.561, 0.657) [*****p*** **< 0.001]**	**0.904 (0.821, 0.987) [*****p*** **< 0.001]**	**0.101**
Outpatient	**0.185 (0.051, 0.319) [*****p*** **= 0.007]**	**0.567 (0.437, 0.696) [*****p*** **< 0.001]**	**0.859 (0.69, 1.03) [*****p*** **< 0.001]**	**0.023**
Emergency room	**0.723 (0.605, 0.84) [*****p*** **< 0.001]**	**0.391 (0.29, 0.493) [*****p*** **< 0.001]**	**1.1 (0.952, 1.24) [*****p*** **< 0.001]**	**0.025**
Inpatient	**0.751 (0.614, 0.887) [*****p*** **< 0.001]**	**0.348 (0.26, 0.435) [*****p*** **< 0.001]**	**1.04 (0.899, 1.18) [*****p*** **< 0.001]**	**0.023**
Prescription	**0.377 (0.316, 0.439) [*****p*** **< 0.001]**	**0.8 (0.753, 0.847) [*****p*** **< 0.001]**	**1.19 (1.12, 1.26) [*****p*** **< 0.001]**	**0.131**
Dental	**−0.686 (− 0.785, − 0.587) [*****p*** **< 0.001]**	**0.154 (0.068, 0.24) [*****p*** **< 0.001]**	**− 0.196 (− 0.332, − 0.061) [*****p*** **= 0.005]**	**0.062**
Home healthcare	**0.543 (0.434, 0.653) [*****p*** **< 0.001]**	**0.316 (0.249, 0.384) [*****p*** **< 0.001]**	**0.642 (0.525, 0.758) [*****p*** **< 0.001]**	**0.064**
Other	**0.219 (0.124, 0.313) [*****p*** **< 0.001]**	**0.505 (0.434, 0.576) [*****p*** **< 0.001]**	**0.763 (0.631, 0.896) [*****p*** **< 0.001]**	**0.02**
Total	**0.443 (0.388, 0.498) [*****p*** **< 0.001]**	**0.625 (0.586, 0.663) [*****p*** **< 0.001]**	**1.04 (0.978, 1.09) [*****p*** **< 0.001]**	**0.142**

**Table 8:** R-square measures for each model for the association between healthcare expenditure and body mass index

Expenditure	Overweight	Obese	R-squared
Office	−0.012 (− 0.061, 0.036) [*p* = 0.616]	0.25 (0.196, 0.304) [*p* < 0.001]	0.09
Outpatient	0.032 (− 0.054, 0.118) [*p* = 0.465]	0.331 (0.241, 0.422) [*p* < 0.001]	0.019
Emergency room	−0.123 (− 0.198, − 0.048) [*p* = 0.001]	0.014 (− 0.061, 0.089) [*p* = 0.713]	0.014
Inpatient	−0.03 (− 0.106, 0.045) [*p* = 0.427]	0.131 (0.059, 0.203) [*p* < 0.001]	0.015
Prescription	0.173 (0.129, 0.218) [*p* < 0.001]	0.563 (0.514, 0.612) [*p* < 0.001]	0.113
Dental	−0.093 (− 0.17, − 0.016) [*p* = 0.018]	−0.25 (− 0.334, − 0.166) [*p* < 0.001]	0.06
Home healthcare	−0.191 (− 0.242, − 0.141) [*p* < 0.001]	−0.032 (− 0.085, 0.021) [*p* = 0.231]	0.057
Other	−0.018 (− 0.083, 0.047) [*p* = 0.588]	0.168 (0.104, 0.233) [*p* < 0.001]	0.014
Total	0.028 (− 0.01, 0.065) [*p* = 0.151]	0.295 (0.253, 0.336) [*p* < 0.001]	0.118

**Table 9:** R-square measures for each model for depression among obese patients with cardiovascular conditions

Expenditure	Unrecognized depression	Asymptomatic depression	Symptomatic depression	R-squared
Office	0.262 (0.151, 0.372) [*p* < 0.001]	0.66 (0.594, 0.726) [*p* < 0.001]	0.976 (0.876, 1.08) [*p* < 0.001]	0.113
Outpatient	0.07 (− 0.119, 0.259) [*p* = 0.47]	0.543 (0.383, 0.703) [*p* < 0.001]	0.863 (0.617, 1.11) [*p* < 0.001]	0.029
Emergency room	0.59 (0.425, 0.755) [*p* < 0.001]	0.312 (0.172, 0.452) [*p* < 0.001]	0.991 (0.798, 1.18) [*p* < 0.001]	0.025
Inpatient	0.578 (0.386, 0.769) [*p* < 0.001]	0.276 (0.16, 0.392) [*p* < 0.001]	0.901 (0.677, 1.13) [*p* < 0.001]	0.022
Prescription	0.368 (0.278, 0.459) [*p* < 0.001]	0.813 (0.751, 0.875) [*p* < 0.001]	1.17 (1.07, 1.27) [*p* < 0.001]	0.155
Dental	−0.583 (− 0.732, − 0.433) [*p* < 0.001]	0.203 (0.079, 0.327) [*p* = 0.001]	0.011 (− 0.156, 0.178) [*p* = 0.899]	0.064
Home healthcare	0.32 (0.185, 0.454) [*p* < 0.001]	0.24 (0.153, 0.326) [*p* < 0.001]	0.533 (0.394, 0.673) [*p* < 0.001]	0.069
Other	0.044 (− 0.08, 0.167) [*p* = 0.487]	0.44 (0.338, 0.543) [*p* < 0.001]	0.782 (0.612, 0.953) [*p* < 0.001]	0.022
Total	0.369 (0.284, 0.454) [*p* < 0.001]	0.601 (0.546, 0.655) [*p* < 0.001]	1.01 (0.935, 1.09) [*p* < 0.001]	0.156

## Abbreviations

BMI: Body Mass Index
CI: Confidence Interval
CPI: Consumer Price Index
ICD-10: International Classification of Diseases, Tenth Revision
ICD-9-CM: International Classification of Disease Version-9-Clinical Modification
MEPS: Medical Expenditure Panel Survey
PHQ-2: Patient Health Questionnaire-2
RECORD: REporting of studies Conducted using Observational Routinely-collected Data
STROBE: STrengthening the Reporting of OBservational studies in Epidemiology
US: United States of America
